# Can Social Motivators Improve Handwashing Behavior among Children? Evidence from a Cluster Randomized Trial of a School Hygiene Intervention in the Philippines

**DOI:** 10.4269/ajtmh.20-0174

**Published:** 2020-11-23

**Authors:** Qayam Jetha, Clément Bisserbe, Jeffery McManus, Daniel Waldroop, Ella Cecilia Naliponguit, Jon Michael Villasenor, Louise Maule, Lilian Lehmann

**Affiliations:** 1 Center for Education Policy Research, Harvard University, Cambridge, Massachusetts;; 2 IDinsight;; 3 Department of Education, Pasig, Philippines;; 4 UNICEF Philippines Country Office WASH, Manila, Philippines

## Abstract

This study reports the impact of the HiFive program, a 6-week handwashing campaign that targets social and emotional motivators to improve student handwashing in primary schools in the Philippines. We designed a clustered randomized trial to evaluate the impact of HiFive on student handwashing behavior, motivation, and access. Of the sample of 196 primary schools located in two districts, half were randomly assigned to receive the program in the 2017–2018 school year. Survey and observation data were collected 3 months after the conclusion of the campaign. In control schools, only 2.5% of students were observed washing their hands with soap and water, our primary outcome and 14.8% were observed washing their hands with at least water. HiFive led to a 3.7 percentage point (p.p.) increase (*P* < 0.01) in the rate of handwashing with soap and water and a 5.6 p.p. increase (*P* = 0.03) in handwashing with at least water after toilet use. HiFive also led to a 10.8 p.p. (*P* < 0.01) increase in the number of handwashing facilities stocked with soap. The program had limited impact on the motivators targeted by the program, suggesting that the small improvements in handwashing may have been driven by increases in the availability of soap. More research is needed to understand how interventions can effectively trigger social motivators to improve handwashing behavior among schoolchildren, and whether the effectiveness of these programs can be augmented with “nudge”-based interventions from the behavioral sciences.

## INTRODUCTION

Evidence linking hand hygiene to reductions in the burden of infectious disease is well-documented. Systematic reviews have found that handwashing with soap (HWWS) reduces diarrheal risk by 31–48%.^1–5^ Although handwashing without soap can significantly reduce bacterial contamination, HWWS is substantially more effective; in one study, HWWS eliminated 71% more bacteria of fecal origin than handwashing with water alone.^6^ Handwashing with soap is also associated with large reductions in other intestinal infections, including parasitic infections as well as respiratory infections, and skin infections.^4,7,8^ Despite the proven health benefits, it is estimated that less than 20% of the world’s population wash their hands with soap after defecating.^3^

A disproportionate share of the burden of infectious disease morbidity and mortality falls on primary school–aged children, and yet in low-income school settings, observed rates of HWWS have been documented to be as low as 2%.^9,10^ Intervening in schools to promote handwashing has been a high priority to break the cycle of disease transmission and realize indirect benefits related to improved student attendance.^11^ Thus far, school hygiene interventions to motivate HWWS have had mixed results, ranging from no effect^10,12–14^ to over 40 percentage points (p.p.) increase in HWWS.^15^ No single approach has emerged as a consistent way to generate large-scale sustained improvements in student handwashing.

Traditional handwashing interventions have focused on addressing supply shortages by improving access to handwashing facilities stocked with soap and increasing demand by educating students on the importance of handwashing.^12,16,17^ However, for children and adults alike, access to facilities coupled with knowledge about the health benefits of handwashing does not always translate into handwashing practice.^12,16–19^ More recent interventions pair improvements in access and knowledge with theory-based approaches to behavior change.^14,19–22^ One such approach is the evolutionary-ecological (Evo-Eco) model of the influences and drivers of human behavior.^20,22,23^ The Evo-Eco model classifies determinants of human behavior drawn from evolutionary theory, psychology, and neuroscience frameworks. Among these determinants, the model proposes a list of 15 fundamental motives based on humans’ evolutionary needs for reproduction and survival that drive behavior change.^24^ Of these 15 motives, two of the most commonly used in handwashing promotion campaigns are “affiliation” and “disgust.”^25^

Affiliation is the tendency for humans to conform to reap the benefits of social living. The fear of social exclusion if an individual is seen to be acting in a way that is socially undesirable is a powerful driver of behavior.^25^ Affiliation includes both empirical expectation (“I want to wash my hands because everyone else washes their hands”) and normative expectation (“I want to wash my hands because I think that other people believe that I should wash my hands”). Disgust is the tendency to avoid something unpleasant or offensive and is thought to be a genetically hardwired emotion that functions as a parasitic avoidance strategy.^25,26^ Disgust can manifest as an individual-level cue (“I want to wash my hands before I eat because if I do not it would be like I am eating feces”) and as a social-level cue (“I want to wash my hands because otherwise I might smell, and others will distance themselves from me”).

Findings from three community-level campaigns targeted at caregivers of children in India, Nepal, and Zambia suggest that using social motivators from the Evo-Eco theory may be an effective strategy to change behavior if the intervention is focused on a single health behavior.^20,27,28^ At the school-level, prior campaigns that harness disgust and affiliation as social motivators of behavior change have had mixed success in improving hand hygiene. The 4-week “School of Five” campaign in Bihar engaged students using posters, diaries, flipchart presentations, and demonstrations to teach children the importance of HWWS, remind children to practice safe hygiene, and build peer pressure around handwashing using the fear of contamination and disgust. Observation data collected 8 weeks following implementation found only a small impact on HWWS after defecation and before eating. Ineffective delivery of key messages, a lack of emotional response to messaging, low campaign intensity, and social and cultural factors were identified as possible reasons for the muted program impact.^14^ Conversely, the Povu Poa pilot study in Western Kenya highlighted more promising results. In addition to providing handwashing stations to public schools, this single-day intervention included activities in the form of skits, songs, and a public handwashing pledge to create a social norm around handwashing and to use disgust as an emotional driver for handwashing. Observed HWWS was found to be 26 p.p. higher in schools that had received the intervention than in schools that had not yet received the Povu Poa program.^21^ However, it is important to note that in this context, baseline availability of soap and water was nonexistent, and it is unclear how much of the change in handwashing was attributable to increased student motivation based on social messaging as opposed to the provision of handwashing stations.

In this study, we report the results of a large-scale, school-based campaign that triggers feelings of disgust and affiliation to motivate student handwashing. At the beginning of the 2017–2018 and 2018–2019 school years, the Philippine Department of Education (DepEd), in collaboration with UNICEF and the International WaterCentre (IWC), implemented a 6-week behavior change campaign, known as the HiFive for Hysan (Hygiene and Sanitation) program, in primary schools across two provinces in the Philippines. We randomly assigned schools to receive the program in either 2017–2018 or 2018–2019 and exploit this phased-in design to measure the impact of the campaign on rates of student handwashing.

## MATERIALS AND METHODS

### Program description.

The HiFive intervention was designed to promote student handwashing after toilet use and before eating. The program was intended to be a supplement to the Philippine water, sanitation, and hygiene (WASH) in schools (WinS) program, which incorporates skills-based learning on hygiene through daily group handwashing and daily group toothbrushing in elementary schools. HiFive was developed following a research study by the IWC that explored handwashing determinants and barriers in eight primary schools in the Philippines.^29^ Using data from student surveys, observations, and interviews, the IWC found that handwashing rates after toilet use and before eating were low, although most students demonstrated an awareness of the health benefits of hand hygiene and had access to a handwashing station with running water (although soap was frequently not present). Students expressed strong visceral reactions to pictures and vignettes depicting poor hygiene practices of a student in different contexts and demonstrated a strong desire to conform to peer norms. These results suggested that disgust and affiliation could be powerful motives for handwashing in this context.

This formative research informed the following set of design principles for a student handwashing intervention:

1. Be based on Evo-Eco motives for behavior change, primarily disgust and peer affiliation.
2. Include messaging on germ transmission to strengthen the effect of emotional and social motives.
3. Leverage student demand for HWWS to improve soap availability and handwashing access.
4. Include fun and engaging activities that can be easily integrated into existing curricula.

**Figure 1.**
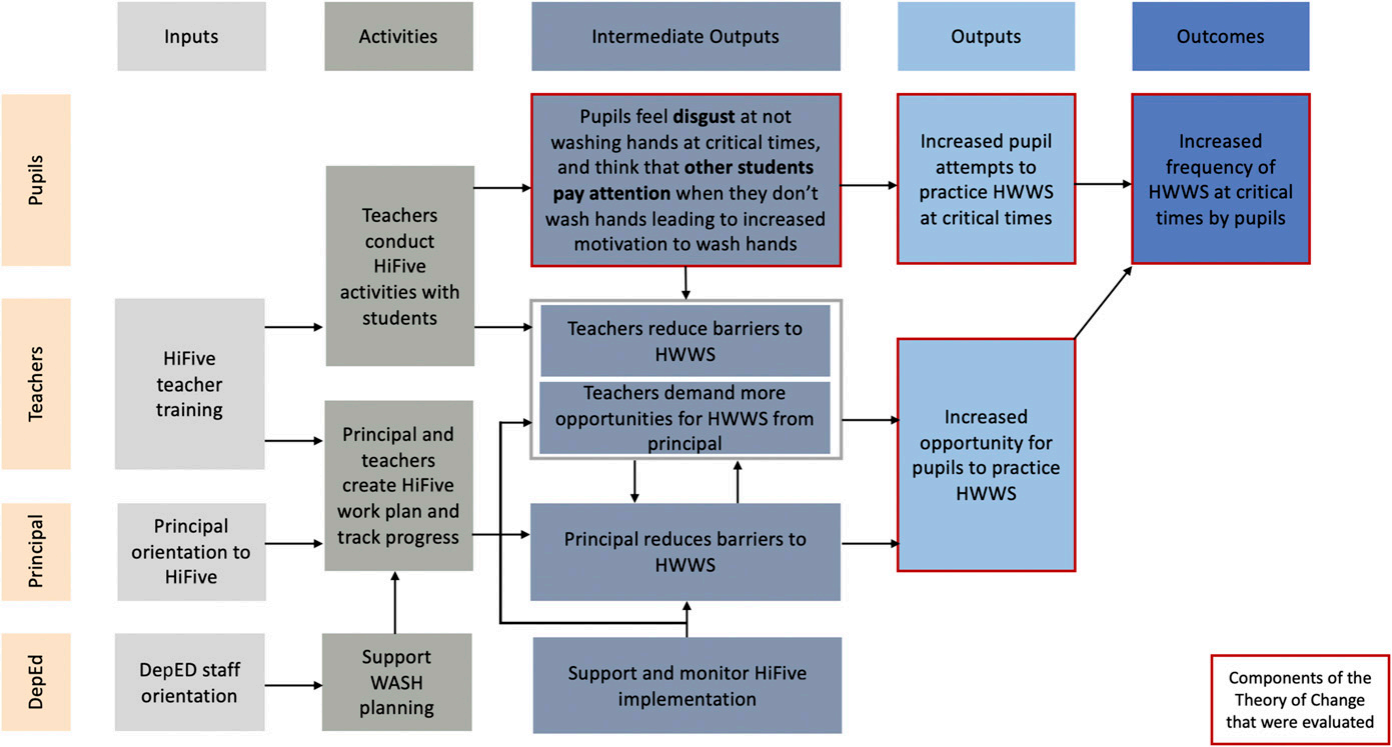
HiFive theory of change. This figure appears in color at www.ajtmh.org.

Based on these design principles, the IWC and UNICEF collaborated to create the HiFive intervention. HiFive was a 6-week school campaign where teachers used a set of HiFive tools (Table 1) to conduct hygiene and sanitation activities (Table 2). The expectation was that the behavioral messaging of HiFive activities would lead to increased motivation for HWWS, increased practice, and, ultimately, increased demand for opportunities to hand-wash. Increased student demand would in turn lead to teachers addressing barriers to HWWS such as ensuring the stocking of soap, while lobbying principals to address more structural barriers to handwashing (see Figure 1 for the HiFive theory of change). The intervention itself did not directly supply soap or handwashing infrastructure to schools.

**Table 1:** HiFive tools

HiFive tool	Description
Storyboard	An interactive flipchart story of two Filipino schoolchildren that builds messages of disgust and affiliation as motivators for handwashing with soap.
Poo-tag	A game to teach students about fecal transmission. Students acting as contaminators try to spread their germs by “contaminating” (tagging) their fellow classmates, while a group of students acting as soap “handwashes” (untags) contaminated students.
iWash song	A song reinforcing messages of the storyboard to be sung during daily group handwashing and after conclusion of poo-tag.
Murals	Scenes from the storyboard are painted as murals in schools to provide visual reminders of key sanitation messages.
Star chart	A chart that maps HiFive activities and other WASH steps that students pledge to complete to meet minimum hygiene and sanitation standards. The chart tracks classroom progress on WASH achievements and serves as a reminder for students about their role in championing hygiene and sanitation at their school.

WASH = water, sanitation, and hygiene

**Table 2:** HiFive activities by campaign week

Week	Activities
1	Storyboard is introduced in science and health and English language classes
	Students are encouraged to call out or remind friends and family who do not wash their hands with soap after using the toilet or before eating
2	Teachers reinforce messages on the benefits of handwashing
	Poo-tag game integrated into PE and science and health classes
	iWash song sang in mother tongue for younger children and in English for older students
	Murals are painted
3	Pupils begin using a toilet worksheet, a supporting tool that encourages pupils on toilet cleanliness
	Teachers propose a schedule for toilet cleaning based on the worksheet
4	Pupils reenact or draw pictures of the storyboard
	Different classes come up with tunes for the iWash song
	Pupils play poo-tag during physical education class
5	In science and health classes, pupils appraise their water, sanitation, and hygiene success using the star chart and identify next steps
	Pupils make the pledge to become HiFive champions by promising to wash their hands with soap
	Award presented for the best storyboard drawing or reenactment is given
6	The campaign closes with a flag ceremony involving Department of Education staff and municipal administrators

### Study design.

To test proof of concept, the DepEd conducted a 2-year pilot study of the HiFive program. Across the 2017–2018 and 2018–2019 school years, HiFive was introduced to a group of primary schools in the school districts of Camarines Norte, in Region V of the Philippines, and Puerto Princesa, located in the island of Palawan in Region IVB. These pilot study locations were selected on account of being UNICEF program areas. Implementation of the first phase of the pilot study took place during the start of the 2017–2018 school year and lasted for 6 weeks from July to August.

We designed a cluster randomized controlled trial to measure the effects of the HiFive intervention on our primary outcome: student HWWS after toilet use, as observed during classroom observations. We also measured the effects of the HiFive intervention on four secondary outcomes:

1. student handwashing with at least water after toilet use, as observed during classroom observations,
2. student handwashing before eating, as reported in student surveys,
3. student handwashing motivations and attitudes, as reported in student surveys,
4. the availability of functional handwashing facilities with soap, as observed during facility spot checks

To construct the evaluation sample of primary schools taking part in the HiFive pilot study, we imposed a set of eligibility criteria based on basic WASH infrastructure requirements (Table 3). This ensured that schools participating in the evaluation had a minimum level of WASH infrastructure in place for the HiFive program to be implemented. Administrative data collected by the DepEd were used to assess school eligibility. Of 328 public primary schools in Camarines Norte and Puerto Princesa, 196 met the three inclusion criteria specified in Table 3. All 196 eligible schools were part of the DepEd’s national WinS policy.

**Table 3:** School inclusion criteria for HiFive pilot participation

No.	Category	Criterion	Schools meeting each Criterion (*n* = 328)	
			No.	%
1	Water	Water for cleaning available at least for certain days of the week	301	91.8
2	Sanitation	Overall student-to-toilet seat ratio is 100 or less	304	92.7
3	Hygiene	At least one functional group handwashing facility	215	65.5
Total schools meeting all criteria			196	59.8

Of the 196 schools meeting the eligibility criteria, half were randomly assigned to receive the HiFive program in the first phase of the pilot study during the 2017–2018 school year (the “treatment group”), and the other half were assigned to receive the intervention in the second phase during the 2018–2019 school year (the “control group”). This evaluation tests the intent-to-treat impact of receiving the HiFive intervention during the 2017–2018 school year.

Randomization was stratified by province and school WASH index to ensure that schools with similar WASH characteristics within Camarines Norte and Puerto Princesa were allocated equally among treatment and control groups. This index included 11 indicators relating to school WASH infrastructure and WASH practices, such as the ratio of students to toilets and implementation of WinS programming. Each school was assigned a point value ranging from 0 (low baseline WASH infrastructure and practices) to 11 (high baseline WASH infrastructure and practices), resulting in 24 province-WASH strata.

We implemented school randomization in Stata/IC version 14.0 (StataCorp, College Station, TX). Within each province-WASH stratum, schools were sorted in increasing order on a random number variable, and the top 50% of schools in each stratum were assigned to treatment and the remainder to control. If a stratum had an odd number of schools, then the last school in this randomized order was alternately assigned to treatment and control, resulting in an equal number of treatment and control schools in the sample overall.

### Sample size.

We conducted power calculations to estimate the required sample size to detect a 10 p.p. improvement in HWWS, which we identified in collaboration with UNICEF as a relevant minimum detectable effect size for informing program recommendations. Assuming 80% power, a two-sided alpha of 0.05, an intra-class correlation of 0.30, and 196 clusters, we estimated that we would need to observe at least 20 handwashing opportunities per school, or 3,920 in total. We assumed that enumerators would need to spend approximately 2 hours of observation per classroom per school to achieve this sample; however, in actuality, this assumption was overly conservative.

### Data collection.

Baseline data were not collected because the HiFive program was implemented at the beginning of the school year and it was not possible to observe student handwashing before the start of school. Endline data were collected between November and December 2017, approximately 3 months after the completion of the HiFive program. We selected 3 months after the completion of the program to collect data because we hypothesized that collecting data right at the end of the program, when the messaging was most salient, could overestimate the medium-term effects of the program. Sixty local enumerators with prior training in data collection, but who were not involved in program implementation, were recruited and trained on our data collection instruments. Each enumerator conducted the following data collection activities:

1. *classroom observations* of student handwashing behavior after toilet use,
2. *student surveys* of self-reported handwashing behavior before eating and after toilet use as well as student motivation and perception toward handwashing, and
3. *facility inspections* of toilet and handwashing station availability and facility characteristics.

### Classroom observations.

All classrooms in grades one to six were eligible for observation, unless the classroom did not have a functioning toilet visible from inside the classroom (2.6% of classrooms) or the classroom had a handwashing facility inside the toilet but did not have a handwashing facility outside the toilet (8.5% of classrooms). Enumerators used SurveyCTO digital data collection software (Dobility Inc., Cambridge, MA) and a complete listing of eligible classrooms to randomly select one classroom in each grade for observation (excluding classrooms that did not have observable handwashing stations). During each observation, enumerators spent 2 hours recording students’ handwashing behavior. While the teacher and students were aware of enumerators’ presence, enumerators explained that they were there to observe “normal classroom activities” and made no mention of handwashing or sanitation. Enumerators chose a discrete spot in the back of the classroom from which to observe without drawing attention to themselves. Each time a student used the toilet, the enumerator noted whether the student washed his or her hands at a handwashing facility immediately after exiting the toilet and whether the student used soap. All students whose handwashing behavior was observed became part of the classroom observation sample.

### Student surveys.

Because students ate lunch at different times and different locations, including at their homes, we were unable to observe whether students washed their hands before they ate lunch. As a result, we relied on surveys of students in grades four to six to measure student self-reported rates of handwashing before eating; younger students in grades one to three were not surveyed because of difficulties in understanding survey questions and remaining focused for the duration of the survey. In each school, two classroom sections per grade were randomly selected for surveying. Enumerators handed out consent forms to students in selected classrooms during their first school visit. Students brought the consent forms home to get a parent or guardian’s signature. Approximately 1 week later, enumerators returned to the school, collected signed consent forms, and randomly sampled eight students per grade, stratified by gender, from selected classrooms using the list of students who returned consent forms.

To obtain the most credible possible estimates from student surveys, we attempted to triangulate handwashing before eating using three survey techniques. The first method was a script recall question to test whether students mentioned HWWS when asked to recount the events that took place between the start of their lunch break and the time they started eating lunch. The second method was a list randomization question as a way for students to report handwashing behavior without allowing the enumerator to identify individual survey responses.^30^ In list randomization, surveyed students received a list of yes/no questions and were asked to respond with the total number of questions (not which ones) that they would respond “yes” to. Thirty percent of the students in our sample (across both treatment and control groups) were randomly selected to receive a four-item question list without the sensitive question asking about handwashing behavior. The other 70% received the same four-item list in addition to the sensitive question on handwashing behavior (for a total of five items). The list randomization impact estimate is the double difference of the average number of items between the treatment group receiving the five-item list and the treatment group receiving the four-item list, and the control group receiving the five-item list and the control group receiving the four-item list. The third and final method that we used to measure handwashing before eating lunch was to directly ask students whether they had done so (direct response).

### Facility inspections.

Using a structured checklist, enumerators conducted inspections of all unlocked student-accessible handwashing facilities within and outside classrooms, checking whether facilities had running water and soap available at the time of inspection.

### Data analysis.

The analytical model was defined before data collection and posted along with the evaluation preregistration on the Registry for International Development Impact Evaluations (study ID: RIDIE-STUDY-ID-5a12613323ff9). Impact estimates reported in the following section are from regression specifications, as described in our pre-analysis plan. Continuous outcomes are modeled using ordinary least squares. Binary outcomes are modeled using linear probability models, which we favored over nonlinear binary choice models, given the coefficient’s direct interpretation as the mean marginal effect of the HiFive intervention. In the context of experimental evaluations, linear probability models provide unbiased estimates of program impact and correctly estimate standard errors.^31^ As a sensitivity check, we have compared linear probability model results with estimated average marginal effects from logistic regressions and found that results do not deviate meaningfully between the two models.

All regressions include school district, WASH index, and strata-level fixed effects, in addition to other control variables specific to the model to reduce variance in the outcome. Standard errors are clustered at the school level, the level of treatment assignment. Sample weights equal to the inverse probability of a classroom section being selected for observation are included for outcomes from classroom observations. For outcomes from student surveys, we include sample weights equal to the inverse of the joint probability of a classroom section being selected for student interviews and a student being selected out of those who returned signed parental consent forms. As shown in the Supplementary Appendix, point estimates are comparable without controls and with equal sample weighting (Section A-2).

*P*-values from regressions are corrected to account for the family-wise error rate using the Holm–Bonferroni method of multiple hypothesis corrections.^32^ Although the pre-analysis plan specified a correction for multiple hypothesis, we deviated from the pre-defined process after further methods research. Specifically, to avoid overcorrection of *P*-values, we separated out the primary outcome (HWWS, as specified in the pre-analysis plan) from secondary outcomes and subgroup analyses in the family-wise adjustments. Furthermore, outcome families with fewer than four outcomes were not corrected because the correction involved a relatively marginal adjustment that would not affect interpretation of results. We include a completed consolidated standards of reporting trials (CONSORT) checklist for cluster randomized trials for this article in the Supplemental Appendix Section A-3.

### Ethical approval.

Before observing classrooms, we received verbal consent from the school principal and teacher. For student surveys, we obtained signed consent from parents and verbal consent from respondents. The stated purpose of our research communicated during the consent process was to observe and learn about student health. Our evaluation was granted ethical clearance from a research ethics committee accredited by the Philippine Health Research Ethics Board—St. Cabrini Medical Center and the Asian Eye Institute—in addition to research clearance from the DepEd.

## RESULTS

### Participants and baseline balance.

Although we were unable to collect student-level data before HiFive implementation, we used pre-program school-level monitoring data on WASH indicators collected by the DepEd to assess baseline balance across our sample of 196 primary schools. As expected, given the stratified randomization performed on an index of WASH characteristics, baseline WASH infrastructure and school hygiene practices did not differ significantly across treatment and control schools (Table 4).

**Table 4:** Balance check and comparison of means across treatment and control schools

	Treatment school mean	Control school mean
Strata variables		
Schools located in Camarines Norte	66.33	66.33
WASH index (out of 11 points)	6.54	6.61
School-level variables		
No. of toilets	12.99	13.24
No. of nonfunctional toilets	0.13	0.13
No. of students	474.98	489.13
Has lunch in canteen (yes/no)	0.85	0.81
Water always available (yes/no)	0.57	0.55
Has regular supply of soap (yes/no)	0.50	0.47
Students bring their own soap (yes/no)	0.21	0.29
HW facility with soap in playground (yes/no)	0.22	0.29
Toilets are cleaned daily (yes/no)	0.92	0.88
No. of teacher-supervised group handwashing events per week	3.33	3.00
No. of teacher-supervised group toothbrushing events per week	2.49	2.87

WASH = water, sanitation, and hygiene. Notes: 1) The school-level variables listed in the balance check table do not include the variables that form the aggregate WASH index. 2) Standard errors are heteroscedasticity robust. 3) The *P*-value from a joint test of orthogonality of all the balance check variables was 0.49.

Of the 196 schools, 12 schools were unreachable for student observations and four schools were unreachable at the time of student surveying. This was on account of unexpected school closures due to holidays, school events, and severe weather. The observation and survey samples remained balanced on baseline school characteristics.

Despite reaching a fewer number of schools, we were able to observe more handwashing opportunities than the required sample size numbers we had estimated. In total, enumerators observed 5,296 handwashing opportunities (2,833 in treatment and 2,463 in control) from 840 classroom observations (438 in treatment and 402 in control). Overall, 4,346 students aged nine to 12 years were selected for surveying from grades four to six; however, 11 students did not provide verbal consent and 40 student surveys were thrown out because of unusually short durations leaving a final sample of 4,295 surveys (2,219 treatment and 2,076 control). Finally, 4,187 handwashing facilities were inspected (2,050 treatment and 2,137 control).

### Intervention compliance.

At the time of data collection, we administered a short survey to teachers and interviewed school administrators to examine the extent that HiFive was implemented with fidelity. Over 90% of teachers surveyed reported attending the 1-day training on HiFive conducted by the DepEd although many cited insufficient training time as a barrier to fully understanding instruction. As a result, HiFive implementation was hindered by teachers’ confusion over the objectives of specific activities and inadequate planning to provide requisite materials. Despite these challenges, teachers largely reported using most of the HiFive tools and most were able to correctly identify the key messages of each activity.

### Handwashing after toilet use.

The overall rate of observed student handwashing in our sample was strikingly low. In control schools, students were observed washing their hands after using the toilet with at least water 14.8% of the time, and with soap only 2.5% of the time.

The HiFive program increased the frequency of handwashing after toilet use; however, the program failed to bring about large-scale hand hygiene behavior change. Treatment students were 3.7 p.p. more likely to wash their hands after using the toilet with water and soap than control students (*P* < 0.01), and 5.6 p.p. more likely to wash their hands with at least water (*P* = 0.03) (Table 5).

**Table 5:** Observed rates of student handwashing after toilet use

	With soap and water	With at least water
School assigned to the HiFive program	0.037***	0.056**
	(0.013)	(0.026)
*R*^2^	0.062	0.085
Observations	5,296	5,296
Control mean	0.025	0.148

*P < 0.10, **P < 0.05, *** *P* < 0.01. Notes: 1) Clustered standard errors are given in brackets. 2) In addition to grade, strata, water, sanitation, and hygiene index, and administrative unit fixed effects, regressions include additional controls for student gender, number of students in the school, and number of students in the class.

### Handwashing before eating.

Surveyed students dramatically overreported their handwashing behavior in both treatment and control schools. The difference between self-reported and observed rates of HWWS after toilet use was 74.4 p.p. in treatment schools and 73.7 p.p. in control schools; the small difference in overreporting between treatment and control schools was not statistically significant. It is likely that self-reported handwashing before eating suffers from similar rates of overreporting.

Table 6 presents the results from three measures of handwashing before eating among students in grades four to six: direct response, script recall, and list randomization. When asked directly about handwashing behavior before their most recent school lunch, 79.6% of students in HiFive schools reported washing their hands with soap compared with 75.3% of students in control schools (*P* = 0.03). The script recall question may have been more successful in reducing self-reporting bias: in the control group, only 31.0% of students recounted washing their hands with soap when asked to describe the events preceding lunch. Students in HiFive schools were 6.4 p.p. more likely to recall washing their hands with soap (*P* = 0.04). The impact estimate from the list randomization method was statistically indistinguishable from zero.

**Table 6:** Student-reported handwashing with soap before eating

	Direct response	Script recall	List randomization
Schools assigned to the HiFive program	0.043** (0.020)	0.064** (0.031)	−0.029 (0.114)
*R*^2^	0.04	0.01	0.11
Observations	4,295	4,295	4,295
Control mean	0.753	0.310	0.310

*P < 0.10, **P < 0.05, *** *P* < 0.01. Notes: 1) Clustered standard errors are given in brackets. 2) Regressions include additional controls for respondent gender, grade, number of students enrolled in the school, whether the survey was conducted before lunch, whether the student was part of a classroom that was observed, and a variable describing the timing of surveying relative to facility spot checks.

### Availability of handwashing facilities.

Access to a functional handwashing facility near a toilet was high across all schools, especially relative to the rate of observed handwashing. In total, 90.4% of toilets in control schools had a functional handwashing facility nearby, compared with 95.4% of toilets in treatment schools (*P* = 0.01).

However, handwashing facilities in HiFive schools were more likely to be stocked with soap. Approximately 38.9% of handwashing facilities near toilets in control schools had soap, compared with 49.7% of facilities in treatment schools (*P* < 0.01). Although the proportion of classrooms with a handwashing facility was similar across treatment groups, classrooms in treatment schools were 10.8 p.p. more likely to have a facility containing soap (*P* < 0.01).

While HiFive appears to have increased the incidence of soap stocked at handwashing stations, the absence of soap was not the only barrier to using soap: Among students who washed their hands only with water, soap was available 74.3% of the time, yet students opted not to use it.

### Student handwashing motivations and beliefs.

**Figure 2.**
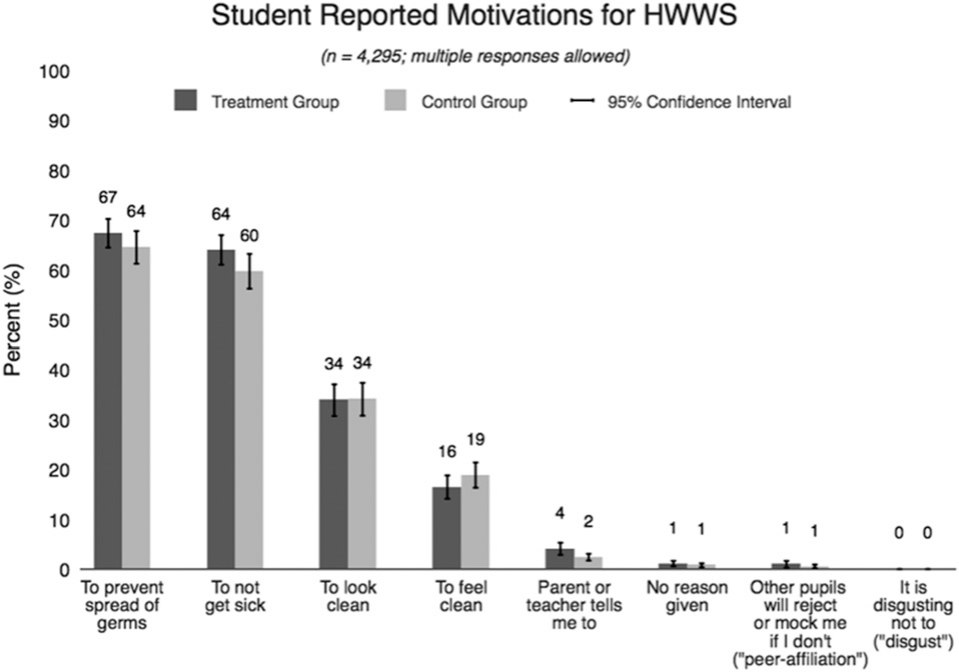
Student handwashing motivations.

Motivations for HWWS reported by students suggest that students were aware of the health benefits of handwashing; the most frequent reasons for handwashing were to prevent the spread of germs (65.9%) and to reduce the incidence of illness (61.8%). However, students rarely responded using visceral language repeated in HiFive program tools such as “gross,” “yuck,” or “eww.” Reasons related to social motivators were also rarely mentioned: approximately 1% of respondents cited other students’ adverse reactions to unwashed hands as a motivator to HWWS (peer affiliation), and there was no difference between treatment and control groups (*P* = 0.91) (Figure 2).

HiFive program tools were designed to strengthen the motivating force of peer affiliation by establishing classroom norms around handwashing. The goal was for students to perceive that their classmates wash their hands with water and soap and that students do not tolerate dirty, unwashed hands. We tested to see if the HiFive program entrenched these norms by asking students whether they believed that their peers wash their hands with soap at critical times (empirical expectation) and whether they believed that students should wash their hands with soap at critical times (normative belief). In total, 25.6% of students in treatment schools reported that every student washes his/her hands with soap after using the toilet and before eating, compared with 22.4% of students in control schools, although the difference was not statistically significant (*P* = 0.16). Similarly, we do not find evidence that the HiFive program led to a notable difference in the proportion of students who strongly believe that other students should wash their hands.

Students who reported not washing their hands were asked to list the reasons why they had neglected to do so. Students were allowed to select multiple reasons. Among the 528 students who did not wash their hands before eating or after using the toilet, the majority said it was because they “forgot” (63%). Other reasons cited by students included that they were in a hurry (24%), reasons related to lack of access to functioning handwashing stations (18%), and because students did not want to (2%).

## DISCUSSION

Three months after the conclusion of HiFive, we find that the program led to a modest gain in the prevalence of handwashing after toilet use. Results are suggestive of similar sized increases in the rate of handwashing before eating. Despite the positive gains, handwashing rates remained extremely low. Student-reported motivations to handwashing provide clues that HiFive did not successfully alter behavioral motivators explicitly targeted by the program. Reasons for handwashing related to peer affiliation and the specific language of disgust used by the HiFive program were rarely mentioned and were not more frequently cited by students in HiFive schools than in control schools. Survey evidence indicates that students’ expectations and normative beliefs around peer handwashing did not change. It is therefore unlikely that HiFive entrenched critical social norms required for peer affiliation to work as a social motivator.

The modest increase in HWWS observed in HiFive schools may be largely attributable to a greater opportunity for HWWS, rather than an increased desire to HWWS. In addition to increasing the presence of handwashing stations nearby toilets, the HiFive program led to sizable increases in the stocking of soap at handwashing facilities and the proportion of classrooms with a handwashing facility containing soap. However, it is unlikely that providing universal access to stocked handwashing facilities would dramatically improve the frequency of student handwashing. In over 90% of classrooms, students had the opportunity to wash hands with water after using the toilet, yet fewer than one in six did so. In more than half of classrooms, students had the opportunity to HWWS, yet the rate of HWWS remained in the single digits. Knowledge gaps did not seem to be the primary constraint. Student surveys indicate high levels of student knowledge on the health benefits of handwashing. This confirms findings from other studies that have shown that access and knowledge by themselves do not translate to sustained large improvements in student handwashing.^12,16–19^

### Limitations of the intervention design.

It is unclear why the HiFive program was unsuccessful in changing students’ motivation for handwashing. One possibility is that a 6-week campaign period may have been too short to lead to sustained behavior change, and students may have required refresher sessions over the school year to reaffirm key HiFive messages. In nonschool settings, successful handwashing promotion interventions are sometimes implemented over a longer time period: a handwashing intervention in Pakistan that halved diarrheal incidence among children involved weekly household visits over the course of a year,^33^ and a handwashing intervention in Ethiopia that reduced parasitic reinfection by half involved weekly household visits over the course of 6 months.^34^ On the other hand, other handwashing interventions have been successful on an even shorter timeline and with fewer sessions than HiFive: the SuperAmma behavior change campaign in India, for instance, involved only 4 days of implementation over the course of a month and led to a 15 p.p. improvement in HWWS.^20^ The Povu Poa pilot study included a single day of behavioral change messaging and found a large impact on observed student handwashing.^21^ Thus, although a longer HiFive intervention period might have further entrenched social norms, we do not regard the 6-week implementation period to be the primary constraint to a greater impact.

Another possibility is that the relevance of social motivators may have been inadequate, given the context and student demographics. Despite formative research suggesting that disgust and affiliation were important motivational drivers among Filipino schoolchildren, we cannot rule out whether other social motivators or a combination of different approaches would have been more effective. For example, some researchers recommend an approach that targets multiple psychological determinants of handwashing at the same time, including motivators that trigger feelings of disgust and social pressure, as well as behavioral nudges that influence automatic or habitual responses.^35–37^

We consider nudge-based interventions to be a particularly promising avenue for promoting handwashing in primary schools. The most often cited reason students gave for not handwashing was because they forgot. Nudges could in theory trigger the automatic psychological responses that counter forgetfulness. In hospitals, universities, and public schools in low-income settings, nudges in public restrooms such as eyes near handwashing stations and arrows pointing from the toilet to the sink have been found to increase rates of HWWS.^38,39^ A proof-of-concept study of a nudge-based intervention targeting handwashing in Bangladeshi schools found that HWWS rates increased 14 p.p. when handwashing infrastructure was moved closer to toilet facilities, but 52 p.p. after a footpath from the toilet to the handwashing facility was painted on the ground.^40^ A follow-up randomized evaluation found a smaller, but sustained, impact of the paved painted footpath and found it to be as effective as a high-intensity hygiene education program.^41^ In Zambian public schools, a simple toilet pass fashioned out of soap attached to a rope was a strong enough contextual cue to motivate student HWWS.^42^

Finally, the HiFive program may have had a sound conceptual basis but was less effective at improving rates of HWWS because of limitations in the implementation design. Specifically, teachers reported that they felt insufficiently trained to deliver all of the activities in the HiFive curriculum. As a result, the HiFive program was overhauled ahead of the second year of implementation to address some of these implementation issues, including further integration of the program’s messaging through detailed lesson plans and more extensive teacher training through a train-the-trainers model. However, handwashing rates among students after implementation of the revamped HiFive program remained below 8%. Given the limited impact of the HiFive program and the effectiveness of behavioral cues in other settings, we are working with UNICEF and the DepEd to design, implement, and evaluate a nudge-based intervention in Filipino primary schools.

### Limitations of the study and areas of further research.

We acknowledge several limitations to our evaluation and suggest topics for further research. First, because the HiFive program appears to have had a limited effect on students’ feelings of affiliation and disgust, we cannot disentangle problems in program delivery from problems with the underlying theory linking social motivators to handwashing behavior; either or both factors may have reduced the effectiveness of HiFive. At the same time, a revamped HiFive program with integrated detailed lesson plans and more extensive teacher training also failed to bring about large-scale behavior change, which makes us somewhat pessimistic about the effectiveness of triggering social motivators in isolation from other interventions. Future research should continue to look at the effect of social motivators, but particularly when complemented with other interventions such as behavioral nudges.

Second, the fact that a majority of students were aware of the importance of handwashing likely led students to overreport handwashing rates in the student survey: students were more than 70 p.p. more likely to say that they washed their hands with soap after toilet use than were actually observed doing so, and it is likely that HWWS before eating was similarly overreported. It is possible that students in treatment schools were more likely to overreport handwashing than students in control schools because of the social desirability effects triggered by HiFive messaging about peer affiliation. For this reason, treatment effects based on self-reported handwashing may be overestimated, and we regard these results as less credible than results based on direct observation.

Social desirability effects may have also led students to wash their hands more in the presence of enumerators than they would otherwise, inflating estimates of HWWS based on direct observation (so-called Hawthorne effects). Although we cannot rule out Hawthorne effects in our study, these effects would have been modest, given the extremely low rates of HWWS overall. We also took several steps to mitigate Hawthorne effects, including conducting direct observation before student surveys about handwashing and giving enumerators a script to explain their presence in the classroom to teachers and students that did not reference handwashing or sanitation. Although we consider it unlikely that large and differential Hawthorne effects biased our evaluation, future research could combine direct observation with other measures, such as measuring microbial contamination of hands or embedding sensors in soap dispensers.

Third, capturing student motivations for performing or failing to perform a specific behavior by asking children to be self-reflective about their actions is likely to be a poor proxy for true motives. Not only is asking these sorts of open-ended questions susceptible to social desirability bias but students may also conflate motives with what they are able to recall at that particular instance in time. As such, we consider self-reported motivations for handwashing from the student survey a relatively weaker form of evidence and encourage future studies to think creatively about ways to use student observation to measure emotions and motivators for handwashing.^43^

Fourth, 39% of classrooms in the study had a handwashing station within the toilet facility that could not be observed by enumerators (in addition to a handwashing station outside the toilet facility); the difference in the fraction of classrooms with a handwashing station within a toilet facility between treatment and control was not statistically significant. The presence of a handwashing station within a toilet facility could lead us to underestimate or overestimate the program effect if students in treatment schools use unobservable handwashing stations at higher or lower rates than students in control school, respectively. Although we find no differential treatment effect on handwashing for students in classrooms that have a handwashing station within a toilet facility versus students in classrooms that do not, we cannot rule out the possibility of either upward or downward bias on our full-sample estimates.

Fifth, we collected data on student handwashing at a single point in time, 3 months after the conclusion of the HiFive intervention. We may have failed to capture larger immediate program effects that tapered off or effects that materialized at a later date. Other studies of handwashing interventions collected outcome data at multiple points during and after the intervention, enabling a more thorough investigation of how treatment effects manifest over time.^8,9,18^ Further research on interventions like HiFive that seek to entrench social norms to improve handwashing behavior may provide valuable evidence on how the effectiveness of these interventions changes over time.

Finally, our study measured the effects of a handwashing intervention targeting social motivators in primary schools across two provinces in the Philippines. To be eligible for our study, schools required functioning handwashing stations and toilet facilities; 40% of schools in the region were not eligible because of a lack of critical handwashing infrastructure. In these schools and other regions, improving WASH infrastructure is a prerequisite to behavior change interventions that target handwashing practices. At the same time, fewer than half of handwashing facilities in our study schools had soap. Further research is needed to evaluate the effectiveness of handwashing interventions that target social motivators in educational settings that start with more and less extensive handwashing infrastructure.

## Supplemental material

Supplemental materials
