# Supplementary material for: Can Social Motivators Improve Handwashing Behavior among Children? Evidence from a Cluster Randomized Trial of a School Hygiene Intervention in the Philippines
Source: Am J Trop Med Hyg. 2020 Nov 23;104(2):756–65. doi: 10.4269/ajtmh.20-0174 (PMC7866363; doi:10.4269/ajtmh.20-0174)
Supplement: Supplementary file 1 [file tpmd200174.SD1.pdf]

Supplemental Appendix to, "Can social motivators improve handwashing behavior among children? Evidence from a cluster randomized trial of a school hygiene intervention in the Philippines", 2020

Contents

Section A-1: Student survey 1
Section A-2: Results 6
Section A-3: CONSORT checklist for cluster randomized trials 15

Section A-1: Student survey

**note_1**	**Section 1: Metadata**	
division	Division	1 Camarines Norte<br>2 Puerto Princesa City
district	Select the District	*District Listing*
school	Select the School	*School Listing*
observer	Enumerator Name	*Enumerator Listing*
date_enum	Enter the date of the survey	
lunch	Is the survey being conducted before or after the pupil's lunch break?	1 Before lunch<br>2 After lunch
grade	What grade is the pupil in?	4<br>5<br>6
section	What section is the pupil in?	
gender	Is the pupil male or female?	0 Male<br>1 Female
assent_script	Hello, my name is [NAME] and I am working on a study conducted by a research organization called IDinsight. The study seeks to better understand pupils' experiences at school, which is why we are interviewing pupils like you. The interview can take up to 15 minutes. I will note down your responses to my questions on this tablet.<br><br>Your responses will be kept confidential and will only be used for research purposes. All personal information will only be seen by staff. You do not need to answer questions if they are uncomfortable to you. You are free to stop the survey at any time.	
consent	Do you agree to participate in this short survey	1 Yes<br>0 No
reason_refused	Please specify the reason why the pupil refused to participate in the survey	
script_recall_instruct_bef<br>[if $lunch==1]	I would like you to tell me what you did during yesterday's lunch break. What time did your morning class end yesterday? And when did you eat? What did you do in between leaving class and eating? Tell me even the small things, like if you ran into a friend, where you ate your lunch, if you ate with anyone, or if you used the CR.	
script_recall_instruct_aft<br>[if $lunch==2]	I would like you to tell me what you did during today's lunch break. What time did your morning class end today? And when did you eat? What did you do in between leaving class and eating? Tell me even the small things, like if you ran into a friend, where you ate your lunch, if you ate with anyone, or if you used the CR.	

script_recall_wash	Did the pupil mention that they washed their hands immediately before eating?	1 Yes<br>0 No
script_recall_soap	Did the pupil mention that they used soap when they washed their hands?	1 Yes<br>0 No
List Randomization "A" Group		
list_rand_a_practice_inst	I am going to ask you 4 "yes" or "no" questions. Put one hand behind your back and count how many questions are "yes" for you. After I ask you all 4 questions, I want you to tell me how many of the questions you answered "yes" to.<br><br>Do you understand the activity?	1 Yes<br>0 No
list_rand_a_practice	1. The last time you came to school, were you on time?<br>2. The last time you had a lunch break, did you buy anything from the canteen or a nearby shop?<br>3. The last time you went to class, did you remember to bring a pencil?<br>4. The last time your school had an athletic competition, did you participate?	
list_rand_a_practice_num	Record the number of "yes" reported by pupil	0<br>1<br>2<br>3<br>4
again_1	I am going to ask you 4 more "yes" or "no" questions. After I ask you all 4 questions, I want you to tell me how many of the questions you answered "yes" to.<br><br>Do you understand the activity?	1 Yes<br>0 No
list_rand_a1_questions	1. The last time you left school, did you leave the campus after 5pm?<br>2. The last time you had recess, did you play any sports?<br>3. The last time you had lunch at the school, did you leave any garbage behind after eating?<br>4. The last time you went to class, did you bring a book-bag?	
list_rand_a1_integer	Record the number of "yes" reported by pupil	0<br>1<br>2<br>3<br>4
again_2	Let's do this one last time.	
list_rand_a2_questions	1. The last time you had a test, did you study the day before the test?<br>2. The last time you had homework, did you finish it on time?<br>3. The last time you had lunch at the school, did you throw away any food you did not want to finish?<br>4. The last time you had recess, did you leave your classroom?	
list_rand_a2_integer	Record the number of "yes" reported by pupil	0<br>1<br>2<br>3<br>4
List Randomization "B" Group		
list_rand_b_practice_inst	I am going to ask you 5 "yes" or "no" questions. Put one hand behind your back and count how many questions are "yes" for you. After I ask you all 5 questions, I want you to tell me how many of the questions you answered "yes" to. I will not know which questions you are saying "yes" or "no" to, I will only ask you for how many questions your answer is "yes", so please feel free to answer each question with the truth. There is no right or wrong answer, I just want to know what your answer is.	1 Yes<br>0 No

	Do you understand the activity?	
list_rand_b_practice	1. The last time you came to school, were you on time?<br>2. The last time you had a lunch break, did you buy anything from the canteen or a nearby shop?<br>3. The last time you went to class, did you remember to bring a pencil?<br>4. The last time your school had an athletic competition, did you participate?<br>5. The last time you went to class, did you quiet down when the teacher told you to?	
list_rand_b_practice_num	Record the number of “yes” reported by pupil	0<br>1<br>2<br>3<br>4<br>5
again_1	I am going to ask you 5 more “yes” or “no” questions. After I ask you all 5 questions, I want you to tell me how many of the questions you answered “yes” to. I will not know which questions you are saying “yes” or “no” to.<br><br>Do you understand the activity?	1 Yes<br>0 No
list_rand_b1_questions	1. The last time you left school, did you leave the campus after 5pm?<br>2. The last time you had recess, did you play any sports?<br>3. The last time you had lunch at the school, did you leave any garbage behind after eating?<br>4. The last time you went to class, did you bring a book-bag?<br>5. The last time you ate lunch on a school day, did you wash your hands with soap immediately before you started eating?	
list_rand_b1_integer	Record the number of “yes” reported by pupil	0<br>1<br>2<br>3<br>4<br>5
again_2	Let’s do this one last time. Remember that you only have to tell me the number of questions you answered “yes” to.	
list_rand_b2_questions	1. The last time you had a test, did you study the day before the test?<br>2. The last time you had homework, did you finish it on time?<br>3. The last time you had lunch at the school, did you throw away any food you did not want to finish?<br>4. The last time you had recess, did you leave your classroom?<br>5. The last time you used a toilet at school, did you wash your hands with soap immediately after using the toilet?	
list_rand_b2_integer	Record the number of “yes” reported by pupil	0<br>1<br>2<br>3<br>4<br>5
direct_response_give	Sometimes, people do not wash their hands with soap and water. Sometimes they may be in a hurry or forget to wash hands. Sometimes there may be no water or soap available.	
direct_response_eating	When you last ate lunch on a school day, immediately before you started eating, did you (a) wash your hands in water (b) wash your hands with water and soap (c) not wash your hands	1 Wash with water<br>2 Wash with soap and water<br>3 No washing
direct_response_toilet	The last time you used a toilet at school, immediately after using the toilet, did you (a) wash your hands in water (b) wash your hands with water and soap (c) not wash you hands	1 Wash with water<br>2 Wash with soap and water<br>3 No washing

why_not_eating<br>[if direct_response_eating == 3]	Why did you not wash your hands before you started eating last time you ate lunch on a school day?	1 No handwashing facility around<br>2 Handwashing facility but no water available<br>3 Handwashing facility but no soap available<br>4 Handwashing facility around but the drain was blocked<br>5 Handwashing facility around, but it was dirty<br>6 I forgot<br>7 I was in a hurry<br>8 I did not want to<br>777 Don't know<br>999 Other
why_not_soap<br>[if direct_response_eating == 2]	Why did you not use soap when you washed your hands before you started eating last time you ate lunch on a school day?	1 No soap available at the handwashing facility<br>2 Soap was available but it was dirty<br>3 I forgot<br>4 I was in a hurry<br>5 I did not want to<br>777 Don't know<br>999 Other
why_not_toilet	Why did you not wash your hands after the last time you used the toilet at school?	1 No handwashing facility around<br>2 Handwashing facility but no water available<br>3 Handwashing facility but no soap available<br>4 Handwashing facility around but the drain was blocked<br>5 Handwashing facility around, but it was dirty<br>6 I forgot<br>7 I was in a hurry<br>8 I did not want to<br>777 Don't know<br>999 Other
why_not_soap2	Why did you not use soap when you washed your hands after the last time you used the toilet at school?	1 No soap available at the handwashing facility<br>2 Soap was available but it was dirty<br>3 I forgot<br>4 I was in a hurry<br>5 I did not want to<br>777 Don't know<br>999 Other
wash_reasons	What are all of the reasons why you wash your hands with soap?<br>*Do not read out options to pupil; select all responses mentioned by pupil; probe by asking "any other reason?" until pupil says no.*	
wash_when	In your opinion, what are the two most important times to wash hands with soap?<br><br>*Read options to pupil out loud.*	0 Never<br>1 Before eating<br>2 After eating<br>3 Before using the toilet<br>4 After using the toilet<br>5 When arriving at school<br>6 Before leaving school

		7 Before arriving home<br>8 After playing<br>999 Other
should_others_toilet	Do you think other pupils in your class should wash their hands with soap immediately after using the toilet?	1 No, definitely not<br>2 No, probably not<br>3 Maybe<br>4 Yes, probably<br>5 Yes, definitely<br>777 I don't know
how_many_others_toilet	In your opinion, out of the pupils in your class, how many of them wash their hands with soap immediately after using the toilet?	1 None of them<br>2 Less than half of them<br>3 Half of them<br>4 More than half of them<br>5 All of them<br>777 I don't know
should_others_eat	Do you think other pupils in your class should wash their hands with soap immediately before eating?	1 No, definitely not<br>2 No, probably not<br>3 Maybe<br>4 Yes, probably<br>5 Yes, definitely<br>777 I don't know
how_many_others_eat	In your opinion, out of the pupils in your class, how many of them wash their hands with soap immediately before eating?	1 None of them<br>2 Less than half of them<br>3 Half of them<br>4 More than half of them<br>5 All of them<br>777 I don't know
ghw	Did you participate in a group handwashing activity with soap supervised by a teacher at school yesterday?	1 Yes<br>0 No
ghw_soap	Did you and the other pupils use soap during the group handwashing activity yesterday?	1 Yes<br>0 No
ghw_num_pupils	How many pupils participated in the group handwashing activity yesterday?	1 All of them<br>2 More than half<br>3 Half<br>4 Less than half
inside_outside	The last time you washed your hands with soap after using the toilet at school, did you wash them inside the toilet facility or at the handwashing facility outside the toilet?	1 Inside the toilet<br>2 Outside the toilet<br>777 Never washed hands with soap after using the toilet at school<br>666 Don't remember
end_note	Thank you very much for answering all of my questions. Now you may return to your class. But before I head back with you, I have a request. May I request that you not tell your classmates what we've talked about, in particular the questions I asked you because I will call one of your classmates to participate and ask these questions to him/her too. And it is important to us that they don't know these questions beforehand. Can I count on you?	
Comments	Do you have any questions or comments for me?	

Section A-2: Results

Table A1: Handwashing with soap after toilet use

ID	*Outcome*	*Sample*	*1. Main Specification (LPM)*	*2. Main Specification (Logit – Average Marginal Effects)*	*3. No controls (LPM)*	*4. Equal weights (LPM)*	*5. No controls, Equal weights (LPM)*
**Main**							
1	Observed HWWS after toilet use *(1=HWWS, 0=No HWWS)*	5,296 students (all grade groups)	.037***	.029***	.027*	.027**	.031**
**Secondary**							
2	Reported HWWS after toilet use, direct response *(1=HWWS, 0=No HWWS)*	4,295 students (grades 4-6)	.045*	.051*	.054*	.027	.028
3	Reported HWWS after toilet use, list randomization *(0-5=number of yes-statements responded in the affirmative, difference-in-differences)*	4,295 students (grades 4-6)	-.050	-	-.038	-.001	.011
**Sub-group analysis of main effect**							
4	Observed HWWS after toilet use by grade group *(grades 1-3 vs. grades 4-6)*	5,296 students (all grade groups)	.013	.020	.007	.012	.010
5	Observed HWWS after toilet use by student gender *(female vs. male)*	5,296 students (all grade groups)	.025	.025	.025	.006	.008
6	Observed HWWS after toilet use by water and soap availability *(available vs. not available)*	5,296 students (all grade groups)	.028	.028	.034	.035**	.037*
7	Observed HWWS after toilet use by WASH index *(0-11 point index)*	5,296 students (all grade groups)	.005	.001	-.002	.005	-.001

8	Observed HWWS after toilet use by number of pupils in classroom *(# of pupils (in '0s))*	5,296 students (all grade groups)	.008	.000	-.002	.004	.000
9	Observed HWWS after toilet use by province *(Puerto Princesa City vs. Camarines Norte)*	5,296 students (all grade groups)	.047*	.031*	-.037	-.012	-.020

(i) "1. Main Specification (LPM)": weights observations by the inverse probability of classroom selection (observed outcomes) or the inverse joint probability of classroom and student selection (reported outcomes). Control variables for regression specifications of observed outcomes include fixed effects for the grade level, district, WASH index, and strata (interaction of division and WASH index), in addition to controls for the number of pupils per school (in '00s), and number of pupils per class. Control variables for regression specifications of reported outcomes include fixed effects for the grade level, district, WASH index, and strata, in addition to controls for the number of pupils per school, gender of student, whether the survey was conducted before lunch, whether the surveyed student was part of a classroom that was observed, and a variable describing the timing of surveying relative to facility observations.
(ii) "2. Main Specification (Logit)": Logit specification run and then Average Marginal Effects are computed using the actual observed values for the variables whose values are not otherwise fixed. Non-linear Difference-in-differences [3] models are not estimated.
(iii) "3. No controls (LPM)": Omits all covariates including strata fixed effects.
(iv) "4. Equal weights (LPM)": Removes probability weights, effectively weighting each observation equally.
(v) "5. No controls, Equal weights (LPM)": Omits covariates and weights each observation equally.
(vi) For all specifications, standard errors are clustered at the school-level.
(vii) Sub-group outcomes were not corrected for multiple inference as all were insignificant.
(viii) Sub-group analysis by province [9] was not specified in the pre-analysis plan.
** p<0.10, **p<0.05, *** p<0.01*

Table A2: Handwashing with at least water (only water or water and soap) after using the toilet

ID	*Outcome*	*Sample*	*1. Main Specification (LPM)*	*2. Main Specification (Logit – Average Marginal Effects)*	*3. No controls (LPM)*	*4. Equal weights (LPM)*	*5. No controls, Equal weights (LPM)*
**Main**							
1	Observed handwashing with at least water after toilet use *(1=HW 0=No HW)*	5,296 students (all grade groups)	.056**	.047**	.018	.059**	.046*
**Secondary**							
2	Reported handwashing with at least water after toilet-use, direct response *(1=HW 0=No HW)*	4,295 students (grades 4-6)	.014	.016	.012	.010	.007
**Sub-group analysis of main effect**							
3	Observed handwashing with at least water after toilet use by grade group *(grades 1-3 vs. grades 4-6)*	5,296 students (all grade groups)	.025	.025	.004	.056	.045

Notes (i)-(vi) in Table A1 apply here.
** p<0.10, **p<0.05, *** p<0.01*

Table A3: Handwashing with soap before eating

ID	*Outcome*	*Sample*	*1. Main Specification* (LPM)	*2. Main Specification (Logit – Average Marginal Effects)*	*3. No controls* (LPM)	*4. Equal weights (LPM)*	*5. No controls, Equal weights* (LPM)
**Secondary**							
1	Reported HWWS before eating, direct response *(1=HWWS 0=No HWWS)*	4,295 students (grades 4-6)	.043**	.045**	.053**	.037**	.036*
2	Reported HWWS before eating, script recall *(1=student mentioned HWWS 0 = student did not mention HWWS)*	4,295 students (grades 4-6)	.064**	.066**	.049	.073**	.058
3	Reported HWWS before eating, list randomization *(0-5=number of yes-statements responded in the affirmative, difference-in-differences)*	4,295 students (grades 4-6)	-.029	-	-.035	-.038	-.053
**Sub-group analysis of direct-response measure**							
4	Reported HWWS before eating, direct response by student gender *(female vs. male)*	4,295 students (grades 4-6)	.052	.054*	.054	.031	.033
5	Reported HWWS before eating, direct response by WASH index *(0-11 point index)*	4,295 students (grades 4-6)	-.001	.005	.002	-.009**	-.005

There is no "main" outcome for student handwashing before eating as hand-washing behavior could not be credibly observed during school lunch time.
Notes (i)-(vi) in Table A1 apply here.
** p<0.10, **p<0.05, *** p<0.01*

Table A4: Handwashing with at least water (only water or water and soap) before eating

ID	*Outcome*	*Sample*	*1. Main Specification (LPM)*	*2. Main Specification (Logit – Average Marginal Effects)*	*3. No controls (LPM)*	*4. Equal weights (LPM)*	*5. No controls, Equal weights (LPM)*
**Secondary**							
1	Reported handwashing with at least water before eating, direct response *(1=HW 0=No HW)*	4,295 students (grades 4-6)	.026**	.032***	.019	0.016	.013
2	Reported handwashing with at least water before eating, script recall *(1=student mentioned HW 0 = student did not mention HW)*	4,295 students (grades 4-6)	0.065*	.072**	.046	.100***	.081*

Notes (i)-(vi) in Table A1 apply here.
** p<0.10, **p<0.05, *** p<0.01*

Table A5: Group handwashing with soap

ID	*Outcome*	*Sample*	*1. Main Specification (LPM)*	*2. Main Specification (Logit – Average Marginal Effects)*	*3. No controls (LPM)*	*4. Equal weights (LPM)*	*5. No controls, Equal weights (LPM)*
**Main**							
1	Observed class conducting a supervised group hand-washing event with soap *(1=GHWWS 0=No GHWWS)*	838 observed classrooms	.034**	.044**	.029**	.032**	.035**
	**Secondary**						
2	Student reported participating in a group hand-washing event with soap in the previous school day *(1=student mentioned GHWWS 0 = student did not mention GHWWS)*	4,295 students (grades 4-6)	.161***	.177***	.177***	.179***	.184***
	**Sub-group analysis of main effect**						
3	Observed class conducting a supervised group hand-washing event with soap by grade group *(grades 1-3 vs. grades 4-6)*	838 observed classrooms	.019	.032	.020	.018	.014

Note that the observed measure was based on an approximate two-hour observation period, whereas the student reported measure asked students about group handwashing over the entire previous school day. Group hand-washing observations were conducted in the 840 classrooms where student hand-washing observations took place. Two classrooms out of the 840 had conflicting reports of group-handwashing and were subsequently dropped, leaving 838 classrooms.
Notes (i)-(vi) in Table A1 apply here.
** p<0.10, **p<0.05, *** p<0.01*

Table A6: Student hand-washing motivations, beliefs, and attitudes

ID	*Outcome*	*Coef. on treatment*	*Std. error*	*p-value*	*Control mean*
**Knowledge of critical times for handwashing, n=4,115**					
1	Student reported before eating as a critical time for handwashing *(1=student mentioned before eating 0=student did not mention before eating, Linear Probability Model)*	.009	.023	.693	.731
2	Student reported after using the toilet as a critical time for handwashing *(1=student mentioned before eating 0=student did not mention before eating, Linear Probability Model)*	.046**	.023	.045	.290
**Reported motivations for handwashing, n=4,295**					
3	To prevent the spread of germs or illness	.010	.023	>.99	.644
4	To stay healthy and not get sick	.062**	.021	.027	.596
5	To look clean	-.022	.024	>.99	.341
6	To feel clean	-.056*	.021	.056	.188
7	Parent or teacher tells me to hand-wash	.013	.014	>.99	.024
8	No reason given	.002	.004	>.99	.008
9	Other students will mock me or reject me if I do not hand-wash	.000	.003	>.99	.005
10	It is disgusting not to hand-wash	.000	.000	>.99	.000
**Student beliefs about other student's hand-washing, n=4,295**					
11	Pupil belief on whether other students wash their hands with soap at critical times – empirical expectation *(1=believe that all students wash their hands before eating and after using the toilet, 0=believe that not every student washes their hands before eating and after using the toilet, Linear Probability Model)*	.032	.023	.161	.224
12	Pupil belief on whether other students should wash their hands with soap at critical times – normative belief *(1=Yes probably or yes definitely, 0=No probably not, no definitely not, or maybe, Linear Probability Model)*	-.006	.020	.770	.849

P-values were corrected for hand-washing motivation outcomes (3-10) using the Holm-Bonferroni correction procedure. Other p-values remain uncorrected.
** p<0.10, **p<0.05, *** p<0.01*

Table A7: Access to hand-washing facilities

ID	*Outcome*	*Sample*	*1. Main Specification (LPM or OLS)*	*2. Main Specification (Logit – Average Marginal Effects)*	*3. No controls (LPM)*
**Availability of hand-washing facilities with soap**					
1	Share of toilets with a nearby functional hand-washing facility with soap *(1=inspected toilet facility has nearby hand-washing facility with soap 0=inspected toilet facility does not have a nearby hand-washing facility with soap)*	2,668 toilets	.109***	.106***	.053
2	Share of inspected classrooms with a functional hand-washing facility with soap *(1=inspected classroom has a functional hand-washing facility with soap 0= inspected classroom does not have a functional hand-washing facility with soap)*	1,780 inspected classrooms	.108***	.105***	.078*
3	Ratio of number of students to the number of hand-washing outlets with soap in the school *(Ordinary Least Squares)*	188 schools	-8.72	-	-11.3
**Availability of hand-washing facilities with or without soap**					
4	Share of toilets with a nearby functional hand-washing facility with or without soap *(1=inspected toilet facility has nearby hand-washing facility with or without soap 0=inspected toilet facility does not have a nearby hand-washing facility with or without soap)*	2,668 toilets	.050***	.047**	.019
5	Ratio of number of students to the number of hand-washing outlets with or without soap in the school *(Ordinary Least Squares)*	188 schools	-4.64	-	-5.56

(i) "1. Main Specification (OLS)": Control variables for regression specifications (3) and (5) include fixed effects for the district, WASH index, and strata (interaction of division and WASH index). In addition to the fixed effects in (3) and (5), regression (2) includes a control for the number of pupils per school. Regression (1) and (4) adds an additional dummy to the covariates in (2) for whether the toilet facility was located inside the classroom.
(ii) "2. Main Specification (Logit)": Logit specification run and then Average Marginal Effects are computed using the actual observed values for the variables whose values are not otherwise fixed.
(iii) "2. No controls": Omits all covariates including strata fixed effects.
(iv) Facility inspection data does not include any sampling weights.
(v) For all specifications, standard errors are clustered at the school-level.
** p<0.10, **p<0.05, *** p<0.01*

Table A8: Availability and condition of toilet facilities

ID	*Outcome*	*Sample*	*1. Main Specification*	*2. No controls*
**Availability of toilet facilities**				
1	Ratio of students to functional toilet facilities *(Ordinary Least Squares)*	188 schools	1.51	1.08
**Condition of existing toilet facilities**				
2	Linear composite toilet facility cleanliness index variable *(Linear composite of the number of following conditions observed in the toilet facility: (a) there is water available for flushing near toilet (b) water in toilet bowl is clear (c) there is a door that can be closed (d) there is a fixed partition, Ordinary Least Squares)*	2,006 toilets	.000	-.031

(i) "1. Main Specification": Control variables for regression include fixed effects for the district, WASH index, and strata (interaction of division and WASH index).
(ii) "2. No controls": Omits all covariates including strata fixed effects.
(iii) Facility inspection data does not include any sampling weights.
(iv) For all specifications, standard errors are clustered at the school-level.
** p<0.10, **p<0.05, *** p<0.01*

Section A-3: CONSORT checklist for cluster randomized trials

Section/Topic	Item No	Standard Checklist item	Extension for cluster designs	Page No *
**Title and abstract**				
	1a	Identification as a randomised trial in the title	Identification as a cluster randomised trial in the title	1
	1b	Structured summary of trial design, methods, results, and conclusions (for specific guidance see CONSORT for abstracts)	See table 2	2
**Introduction**				
**Background and objectives**	2a	Scientific background and explanation of rationale	Rationale for using a cluster design	4-5
	2b	Specific objectives or hypotheses	Whether objectives pertain to the the cluster level, the individual participant level or both	5
**Methods**				
**Trial design**	3a	Description of trial design (such as parallel, factorial) including allocation ratio	Definition of cluster and description of how the design features apply to the clusters	7-8
	3b	Important changes to methods after trial commencement (such as eligibility criteria), with reasons		9 (inaccessibility of 12 of the 196 schools in the sample)
**Participants**	4a	Eligibility criteria for participants	Eligibility criteria for clusters	7,9
	4b	Settings and locations where the data were collected		7-9
**Interventions**	5	The interventions for each group with sufficient details to allow replication, including how and when they were actually administered	Whether interventions pertain to the cluster level, the individual participant level or both	5-6
**Outcomes**	6a	Completely defined pre-specified primary and secondary outcome measures, including how and when they were assessed	Whether outcome measures pertain to the cluster level, the individual participant level or both	7,9-10
	6b	Any changes to trial outcomes after the trial commenced, with reasons		N/A
**Sample size**	7a	How sample size was determined	Method of calculation, number of clusters(s) (and whether equal or unequal cluster sizes are assumed), cluster size, a coefficient of intracluster correlation (ICC or *k*), and an indication of its uncertainty	8
	7b	When applicable, explanation of any interim analyses and stopping guidelines		N/A
**Randomisation:**				
**Sequence generation**	8a	Method used to generate the random allocation sequence		8

	8b	Type of randomisation; details of any restriction (such as blocking and block size)	Details of stratification or matching if used	7-8
**Allocation concealment mechanism**	9	Mechanism used to implement the random allocation sequence (such as sequentially numbered containers), describing any steps taken to conceal the sequence until interventions were assigned	Specification that allocation was based on clusters rather than individuals and whether allocation concealment (if any) was at the cluster level, the individual participant level or both	8
**Implementation**	10	Who generated the random allocation sequence, who enrolled participants, and who assigned participants to interventions	Replace by 10a, 10b and 10c	
	10a		Who generated the random allocation sequence, who enrolled clusters, and who assigned clusters to interventions	8
	10b		Mechanism by which individual participants were included in clusters for the purposes of the trial (such as complete enumeration, random sampling)	9
	10c		From whom consent was sought (representatives of the cluster, or individual cluster members, or both), and whether consent was sought before or after randomisation	9, 11
**Blinding**	11a	If done, who was blinded after assignment to interventions (for example, participants, care providers, those assessing outcomes) and how		N/A
	11b	If relevant, description of the similarity of interventions		N/A
**Statistical methods**	12a	Statistical methods used to compare groups for primary and secondary outcomes	How clustering was taken into account	10-11
	12b	Methods for additional analyses, such as subgroup analyses and adjusted analyses		10-11
**Results**				
**Participant flow (a diagram is strongly recommended)**	13a	For each group, the numbers of participants who were randomly assigned, received intended treatment, and were analysed for the primary outcome	For each group, the numbers of clusters that were randomly assigned, received intended treatment, and were analysed for the primary outcome	9-10
	13b	For each group, losses and exclusions after randomisation, together with	For each group, losses and exclusions for both clusters and individual cluster members	9

		reasons		
**Recruitment**	14a	Dates defining the periods of recruitment and follow-up		8-9
	14b	Why the trial ended or was stopped		9
**Baseline data**	15	A table showing baseline demographic and clinical characteristics for each group	Baseline characteristics for the individual and cluster levels as applicable for each group	8
**Numbers analysed**	16	For each group, number of participants (denominator) included in each analysis and whether the analysis was by original assigned groups	For each group, number of clusters included in each analysis	Tables 5 & 6, Figure 1
**Outcomes and estimation**	17a	For each primary and secondary outcome, results for each group, and the estimated effect size and its precision (such as 95% confidence interval)	Results at the individual or cluster level as applicable and a coefficient of intracluster correlation (ICC or k) for each primary outcome	11-14
	17b	For binary outcomes, presentation of both absolute and relative effect sizes is recommended		11-14
**Ancillary analyses**	18	Results of any other analyses performed, including subgroup analyses and adjusted analyses, distinguishing pre-specified from exploratory		Appendix
**Harms**	19	All important harms or unintended effects in each group (for specific guidance see CONSORT for harms)		N/A
**Discussion**				
**Limitations**	20	Trial limitations, addressing sources of potential bias, imprecision, and, if relevant, multiplicity of analyses		15-16
**Generalisability**	21	Generalisability (external validity, applicability) of the trial findings	Generalisability to clusters and/or individual participants (as relevant)	16
**Interpretation**	22	Interpretation consistent with results, balancing benefits and harms, and considering other relevant evidence		14-16
**Other information**				
**Registration**	23	Registration number and name of trial registry		2
**Protocol**	24	Where the full trial protocol can be accessed, if available		2
**Funding**	25	Sources of funding and other support (such as supply of drugs), role of funders		19

CONSORT template downloaded from:
http://www.consort-statement.org/Media/Default/Downloads/Extensions/CONSORT%20Extension%20for%20Cluster%20Trials%202012%20Checklist.docx
